# Evaluation of the efficacy of amplitude-integrated electroencephalography in the screening of newborns with metabolic disorder admitted to the NICU

**DOI:** 10.1186/s12880-018-0274-4

**Published:** 2018-09-20

**Authors:** Maliheh Kadivar, Ziba Mosayebi, Reza Shervin Badoo, Raziyeh Sangesari, Saeed Jedari Attari, Maryam Saeedi, Elahe Movahedi Moghadam

**Affiliations:** Neonatal Health Research Center, Research Institute for Children Health, Shahid Beheshti University of Medical Sciences, Tehran, Iran

**Keywords:** aEEG, Metabolic diseases, Neonates

## Abstract

**Background:**

Neonate patients with metabolic disorder show encephalopathy and seizures that may lead to morbidity and mortality. Thus rapid detection and treatment of these patients is necessary. Although Amplitude-integrated electroencephalography (aEEG) has been used for more than a decade in the evaluation of infants with encephalopathy but has not been used in the assessment of neonates suffering from metabolic disorders. In this study, we tried to determine the efficacy of aEEG as an easily available diagnostic tool in the diagnosis of neonates with metabolic diseases.

**Methods:**

All cases which admitted to the Neonatal Intensive Care Unit (NICU) of the Children’s Medical Center during a one-year period were enrolled. aEEG recordings were obtained by installing 4 electrodes on the infant’s head by a trained nurse and aEEG was recorded for at least 24 h with a description of the whole tracing. Clinical information, final outcome and questionnaires, including patient information: symptoms of the disease, gender, age, duration of hospitalization and the type of the metabolic disease were recorded in details. The obtained data was analyzed with the Spss24 software.

**Results:**

Only 3 (two girl and one boy) out of 29 aEEGs recordings were abnormal; other patients showed normal aEEGs. The most common clinical and neurological manifestations were seizure (34.5%), hypotonia (31%), and mortality rate was 10.3%. There was no significant correlation between aEEG findings and gender, age, type of disease, laboratory tests findings and positive family history.

**Conclusions:**

Although it has been shown that EEG has a diagnostic value in metabolic diseases, there has been no study on the efficacy of aEEG to evaluate neonates with metabolic diseases. But good accessibility and easy of working with aEEG, promote a tendency to use this procedure as screening tool for metabolic diseases.

The current study about aEEG monitoring in these patients, while limited, can be used as a pilot study for further research on this topic.

Therefore, a correct judgment in this field requires administration of aEEG on a larger population of neonates with metabolic diseases.

## Background

Inherited metabolic disorders are genetic conditions that result in metabolism problems namely a lowered or deficient activity of an enzyme in a single pathway of intermediary metabolism [1]. Due to the problems in metabolism and homeostasis of sugar, electrolytes, amino acids and ammonia, widespread brain dysfunction may occur in these patients [2].

Metabolic disorders in newborns can ultimately lead to problems such as seizure, mental retardation and even death [3]. Thus screening of metabolic disorders in newborns helps to the earliest possible diagnosis and management of the affected newborns, which in turn prevents the morbidity, mortality, and disabilities associated with the metabolic disorders [1].

Metabolic disorders screening is usually performed by means of various clinical analysis on blood and urine samples as well as imaging evaluations [4]. On the other hand, metabolic disorders are not symptomatic immediately after the birth and they can even manifest slowly with progressive encephalopathy [5]. Thus accurate diagnostic evaluation of neural function in newborns with a possible metabolic disorder is very important [4].

Electroencephalography (EEG) is one of the required tests in order to diagnose and assess the clinical condition of metabolic disorders through the abnormalities in brain electrical activity [6]. Although EEG is a non-invasive test that helps to determine metabolic diseases, the complexity of the equipment and the need for specialized staff to apply the numerous electrodes make it difficult to use.

In contrast, an appropriate alternative to solve this problem in Neonatal Intensive Care Units (NICUs) is the amplitude integrated electroencephalography (aEEG). According to previous studies, in order to generate an aEEG, one or two-channel EEG tracings are obtained, filtered, rectified and compress in time to provide a global overview of brain cerebral activity [7, 8]. Notably, frontal leads are less commonly used to detect seizures because, according to the literature, it is possible to miss more pathologies over frontal region. The 2-channel aEEG from channel C3 to P3 and C4 to P4 (named by the 10–20 system) is able to identify unilateral pathologies in brain activity. Additionally, more pathologies can be missed with a 1-channel aEEG as compared to the 2-channel method [9]. Applying the aEEG is inexpensive, easy and affordable procedure that could be carried out by NICU staff after a fairly short training period [7]. Today, aEEG is used routinely in an increasing number of NICUs, as a quantitative predictor and provides a global overview of brain cerebral activity [8, 10].

Studies in the current literature show that aEEG could help to diagnose and follow up the newborns with neonatal encephalopathy [6, 11, 12]. Although studies show the benefits of EEG for initial evaluation and management of patients with metabolic disorder [6, 13], no study has been conducted to assess the efficiency of aEEG for the assessment of newborns with metabolic diseases.

Therefore, in this study, we tried to determine aEEG performance in the diagnosis of neonates with metabolic diseases through diagnosing abnormalities in electrical brain activity.

## Methods

The present study was conducted over a one-year period (April, 2017-April, 2018) on neonates with suspicious metabolic disorders who were admitted to the NICU of the Children’s Medical Center, Tehran, Iran.

The study proposal was approved in the local ethics committee and informed consents were obtained from parents of neonates who fulfilled the inclusion criteria. Inclusion criteria included neonates with:

### Laboratory criteria

- Ammonia > 150 μg/dl
- Lactate > 30 mmol/l
- Blood Sugar (BS) < 40 mg/dl
- High-anion gap acidosis resistant to treatment, and Base Excess (BE) < − 10
- HPLC (High-performance liquid chromatography) results that indicates metabolic disorder
- Glycine of cerebrospinal fluid /serum > 0.08
- Urine findings: Organic urine acid, regenerative materials and positive ketones
- Electrolyte disorders: hypo / hypernatremia, hyper / hypoglycemia, hyper / hypocalcaemia and hypomania

### Clinical criteria

- Neurological symptoms: seizure, hypertonia, decreased consciousness
- Gastrointestinal symptoms: prolong jaundice, hepatosplenomegaly
- Cardiovascular Symptoms: Cardiomyopathy, hypotension
- Systemic Symptoms: Unwillingness to eat milk, tachypnea, acidosis, especially in cases of familial marriage
- Hydrops
- Positive family history of metabolic disease (previous child)
- Death without known cause
- Failure to justify the above with common diseases (sepsis)

Exclusion criteria included neonates with asphyxia diagnosis, death before aEEG acquisition and parent dissatisfaction.

The aEEG evaluations were done through installing 4 electrodes on the infant’s head (on c3, c4, p3, p4) by a trained nurse and recorded for at least 24 h with a description of the whole tracing. Patients’ diagnoses and relevant clinical information were also recorded. The obtained aEEG recordings were collected and interpreted by both pediatrician and neurologist subspecialist clinicians carefully.

Findings were recorded using questionnaires, including patient information: symptoms of the disease, gender, age, duration of hospitalization and type of metabolic disease.

The final outcome was determined by the length of hospitalization, the response to treatment, discharge from NICU or death.

### Statistical analyses

Descriptive analyses of the data were performed using McNemar test. Pearson’s chi-squared test and independent sample t-test were used to evaluate the relationship aEEG abnormality and different metabolic disorders and the differences between neonates with normal and abnormal aEEG, respectively. The analyses were performed using SPSS software version 24 and a *P* < 0.05 was considered statistically significant.

## Results

A total of 29 patients in NICU department were enrolled in the present study. Of the neonates, 15 (51.7%) were boy and 14 (48.3%) were girl. The mean age of neonates was 10.62 (±5.6) days. Also the mean of gestational age was 37.8 weeks (±0.88).

The most common neurological manifestations were seizure (34.5%), hypotonia (31%), and the mortality rate was 10.3%.

In this study, the most common metabolic diseases in the neonates included hypoglycemia (24.1%), urea cycle (20.7%), and NKH (Nonketotic hyperglycinemia) (17.2%) and the least common were glycine deficiency (3.4%), Zellweger (3.4%) and familial hypercalciuria hypomagnesemia (3.4%).

The results show no significant correlation between aEEG findings (normal and abnormal) and different metabolic diseases (*p* = 0.839) (Table 1). There was no relation between age, familial relation, final outcome, ultrasound result, MS MS result, HPLC result, Ammonia, lactate, ABG amount with aEEG findings.

**Table 1:** Relationship between different metabolic diseases and aEEG/EEG results

Metabolic diseases	EEG			aEEG		
	Abnormal No (%)	Normal No (%)	*P*.value	Abnormal No (%)	Normal No (%)	*P*.value
Hypoglycemia	1 (14.3)	6 (85.7)	0.016	0(0)	7(100.0)	0.839
Urea cyclic disorder	1(16.7)	5(83.3)		1(16.6)	5(83.3)	
Galactosemia	0(0)	3(100.0)		0(0)	3(100.0)	
MSUD	3(100.0)	0(0)		1(33.3)	2 (66.7)	
NKH	4(80.0)	1(20.0)		1(20.0)	4 (80.0)	
Familial hypercalciuria hypomagnesemia	0(0)	1(100.0)		0(0)	1(100.0)	
Zellweger	0 (0)	1 (100.0)		0(0)	1(100.0)	
Glycine deficiency	1 (100.0)	0(0)		0(0)	1(100.0)	
Tyrosinemia	2 (100.0)	0(0)		0(0)	2(100.0)	
Total	12 (41.4)	17 (58.6)		3 (10.34)	26 (89.65)	

However, as shown in the Table 1, there is a significant statistical relationship between EEG findings and the type of metabolic disease in newborns (*p* = 0.016, _χ_2 = 18.7). There was no relation between age, familial relation, final outcome, ultrasound result, MS MS result, HPLC result, Ammonia, lactate, ABG amount with EEG findings.

As shown in Table 2, none of the patients’ clinical characteristics (with or without seizure) were significantly associated with aEEG (*p* = 1) and EEG (*p* = 0.449) results.

**Table 2:** Relationship between EEG and aEEG results with seizure manifestations of the studied neonates

	Seizure manifestations		Total	Pearson chi-square	*P*.value Fisher’s exact test
	YES	NO			
EEG					
Abnormal	9	3	11	0.367	0.449
Normal	10	7	17		
Total	19	10	29		
aEEG					
Abnormal	2	1	3	0.965	1
Normal	17	9	27		
Total	19	10	29		

In this study, we also investigated the relationship between aEEG and EEG in neonates with metabolic diseases. The results showed that none of aEEG findings were significantly associated with EEG results (*p* = 0.553) (Table 3).

**Table 3:** Relationship between EEG results with aEEG results of the studied neonates

	EEG		Total	Pearson chi-square	*P*.value Fisher’ sexact test
	Abnormal	Normal			
aEEG					
Abnormal	2	1	3	0.348	0.553
Normal	10	16	26		
Total	12	17	29		

Finally, the measure of agreement between pediatric neurologists and neonatologist reports based on aEEG findings was 0.724 (strong) and statistically significant (*p* = 0.003) (Table 4).

**Table 4:** Agreement measure between pediatricians and neurologists based on aEEG finding

	Neonatologist			Total	Measure of agreement Kappa	*P*. value
	Continuous normal voltage	Discontinuous normal voltage	Burst suppression			
Neurologist						
Continuous normal voltage	24	0	0	24	0.713	0.003
Discontinuous normal voltage	2	1	0	3		
Burst suppression	0	0	2	2		
Total	26	1	2	29		

## Discussion

In this study we obtained of the 24 h-length aEEG tracings of 29 patients with different metabolic disorders. Among 29 neonates, 41.4% had abnormal EEG and 6.89% showed abnormal aEEG. The findings of aEEG were abnormal in two girls and one boy, and normal in 14 boys and 12 girls. Thus, there was no significant correlation between the findings of aEEG (normal and abnormal) and gender.

In this study, we examined the difference between the type of neonatal metabolic disease and aEEG. Our results showed no significant relation between aEEG finding and type of disease. However, given the sample size and the number of patients in different metabolic disease in this pilot study, we do not expect a meaningful association between aEEG finding and type of disease. Thus it seems that aEEG abnormalities help to monitor brain function in neonates with metabolic disorder rather than diagnostic performance towards the type of metabolic disorder [13]. Our results are in accordance with the results reported by Olischar et al. who showed that aEEG is not ‘diagnostic’ in IEM (Inborn error of metabolism) but it could help in assessing the severity of encephalopathy and seizure [13]. This valuable use of aEEG has been reported by Theda et al. [7] where encephalopathy and seizure were found in IEM patients by aEEG monitoring. Also in another study on a case of hyperammonemic coma, the response to the treatment was assessed by aEEG successfully [11].

In this study, normal EEG results were consistent with normal aEEG in some diseases. For example, normal EEG in urea cycle disorder was found in 100% of cases, which was in accordance with 100% normal aEEG finding. Similarly, in the galactosemia, zellweger and Familial hypercalciuria hypomagnesemia, EEG findings were similar to aEEG findings.

As reported there was no relation between age, familial relation of the parents, final outcome, ultrasound results, MS MS result, HPLC result, Ammonia, lactate, ABG amount and aEEG and EEG findings.

We have also found a significant agreement between pediatric neurologists and neonatologist on the aEEG report. Considering the 93.1% agreement between pediatric neurologists and neonatologist in reading and reporting aEEG, and simplicity of using aEEG by other specialists, it seems that this device could be a good procedure for screening seizure problems in NICUs.

Other studies also reported that aEEG has high sensitivity and specificity in predicting neurodevelopmental outcome for screening of asphyxiated full-term infants [14–17].

## Conclusion

Although finding of this study showed no relation between aEEG and different metabolic disorder, this procedure can provide valuable information about brain activity, especially during metabolic crisis. In regard to the low number of patients in this study, more studies with larger population are needed to determine the amplitude-integrated efficacy of EEG in neonates with metabolic disorder. However, inspite of the above-mentioned limitations, the current study appears to be a promising avenue to perform further projects in this field.

## Abbreviations

aEEG: Amplitude-integrated electroencephalography
BE: Base Excess
BS: Blood Sugar
HPLC: High-performance liquid chromatography
NICU: Neonatal Intensive Care Unit
NKH: Nonketotic hyperglycinemia
